# Deposition of Immune Complexes in Gingival Tissues in the Presence of Periodontitis and Systemic Lupus Erythematosus

**DOI:** 10.3389/fimmu.2021.591236

**Published:** 2021-03-25

**Authors:** Julien Rodrigues Pires, Maria Renata Sales Nogueira, Adauto José Ferreira Nunes, Débora Regina Fernandes Degand, Larissa Costa Pessoa, Carla Andreotti Damante, Mariana Schutzer Ragghianti Zangrando, Sebastião Luiz Aguiar Greghi, Maria Lúcia Rubo de Rezende, Adriana Campos Passanezi Sant'Ana

**Affiliations:** ^1^Discipline of Periodontics, Department of Prosthodontics and Periodontics, School of Dentistry at Bauru, University of São Paulo, Bauru, Brazil; ^2^Lauro de Souza Lima Institute, São Paulo State Health Secretariat, Bauru, Brazil; ^3^Cell Biology Lab, Lauro de Souza Lima Institute Hospital, Bauru, Brazil; ^4^Department of Dentistry, Paulista University, Brasília, Brazil

**Keywords:** systemic lupus erythematosus, periodontitis, immune complex, inflammation, diagnosis

## Abstract

Systemic lupus erythematosus (SLE) is a complex chronic autoimmune disease characterized by tissue damage and widespread inflammation in response to environmental challenges. Deposition of immune complexes in kidneys glomeruli are associated with lupus nephritis, determining SLE diagnosis. Periodontitis is a chronic inflammatory disease characterized by clinical attachment and bone loss, caused by a microbial challenge – host response interaction. Deposition of immune complex at gingival tissues is a common finding in the course of the disease. Considering that, the primary aim of this study is to investigate the deposition of immune complexes at gingival tissues of SLE patients compared to systemically healthy ones, correlating it to periodontal and systemic parameters. Twenty-five women diagnosed with SLE (SLE+) and 25 age-matched systemically healthy (SLE–) women were included in the study. Detailed information on overall patient's health were obtained from file records. Participants were screened for probing depth (PD), clinical attachment loss (CAL), gingival recession (REC), full-mouth bleeding score (FMBS) and plaque scores (FMPS). Bone loss was determined at panoramic X-ray images as the distance from cementenamel junction to alveolar crest (CEJ-AC). Gingival biopsies were obtained from the first 15 patients submitted to surgical periodontal therapy of each group, and were analyzed by optical microscopy and direct immunofluorescence to investigate the deposition of antigen-antibody complexes. Eleven (44%) patients were diagnosed with active SLE (SLE-A) and 14 (56%) with inactive SLE (LES-I). Mean PD, CAL and FMBS were significantly lower in SLE+ than SLE–(*p* < 0.05; Mann Whitney). The chronic use of low doses of immunosuppressants was associated with lower prevalence of CAL >3 mm. Immunofluorescence staining of markers of lupus nephritis and/or proteinuria was significantly increased in SLE+ compared to SLE–, even in the presence of periodontitis. These findings suggest that immunomodulatory drugs in SLE improves periodontal parameters. The greater deposition of antigen-antibody complexes in the gingival tissues of patients diagnosed with SLE may be a marker of disease activity, possibly complementing their diagnosis.

## Introduction

Systemic lupus erythematosus (SLE) is a chronic, autoimmune disease caused by a combination of genetic, environmental and immunologic factors. It is characterized by varying clinical manifestations and by the presence of several autoantibodies, including anti-DNA, anti-nuclear (ANA) or anti-phospholipids antibodies. It affects mostly women at 15–45 years, in a proportion of 10:1 (1, 2). Its incidence is relatively rare, affecting 56 out of 100,000 Americans (3) and 87 out of 100,000 in Brazil (4). Approximately, 1.5 million Americans and 5 million people around the world have lupus (5).

The diagnosis of SLE is complex, since it presents periods of flare and quiescence and a wide range of clinical manifestations, such as malar rash, pain, fatigue, hair loss, physical impairment, seizures or psychosis, anemia, leukopenia or lymphopenia. The American College of Rheumatology (6, 7) and the Systemic Lupus International Collaborating Clinics (SLICC) (8) have defined that ≥4 criteria must be present to determine SLE diagnosis. SLICC classification criteria requires that one clinical and one laboratory criteria must be present or biopsy-proven lupus nephritis with positive ANA or anti-dsDNA antibodies. Diagnosis cannot be made at the onset of symptoms, and long periods of observation are necessary up to new clinical manifestations determine SLE. Immunological findings include depression of complement (low C3 and/or C4 or CH50) and high titers of varying circulating autoantibodies and deposition of immune complexes capable of activating complement and inflammation, resulting in multiorgan damage (9). Recently, a scoring system based on positive history of ANA through Hep-2 immunofluorescence was proposed (10). The production of anti-dsDNA is also considered as a cardinal sign of lupus, as its levels are correlated with disease activity (11).

Lupus nephritis (LN) is characterized by immune aggregates at sites of injury in glomeruli and in the tubules in ~ 2/3 of renal biopsies. These immune complexes may be derived from circulating complexes or from *in situ* combination of antigen and antibody. Usually, patients with lupus nephritis show antibodies against dsDNA, Sm and C1q (12). In clinical practice, it is essential to evaluate patients' kidney status. A renal biopsy is a standard diagnostic tool for the evaluation of kidney lesions in SLE, but due to its invasive nature, a kidney biopsy has potential risks and as a rule, it is not routinely performed (13).

Not all SLE patients develop LN. It is significantly more prevalent especially in blacks, associated to genetic risk factors. LN occurs when the expression of neutrophil-associated genes increases, preceded by upregulation of proinflammatory transcripts. Even after death, neutrophils may contribute to tissue damage through the formation of neutrophils extracellular traps (NETS) that may facilitate inflammation and cause endothelial damage, amplifying autoimmunity (14).

Periodontitis is a chronic infectious and inflammatory disease caused by microbial dysbiosis, characterized by loss of attachment and alveolar bone around natural teeth. Secondary features include pocket formation, bleeding on probing, tooth mobility and suppuration, among others. Histopathological findings of advanced periodontal lesions depicts plasma cells and lymphocytes occupying a vast area of gingival connective tissues and elevated serum titers of IgG against periodontal pathogens, resulting in the formation of immune complex that deposit on gingival tissues (15–20), activating complement and neutrophils, and triggering inflammatory responses (21). Worse severity of periodontitis is associated to an exacerbated inflammatory response to microbial challenge.

The pathogenic mechanisms of periodontitis and SLE presents some similarities, as with other autoimmune diseases (22, 23). Deregulation of immune system, with a key role exerted by neutrophils, phagocytic cells and pro-inflammatory cytokines contribute to tissue destruction in both conditions (24). Recently, our research group showed significant upregulation of serum proinflammatory cytokines in individuals with SLE compared to controls. Anti-inflammatory IL4 and IL-10 were upregulated only in inactive SLE sera, controlling clinical phenotypes. Out of 24 oral microbial abundances, 14 unique subgingival bacteria profiles were elevated at SLE, especially *T. denticola* and *T. forsythia* in active SLE compared to inactive SLE and healthy controls. These findings suggested that low-grade systemic inflammation that influence SLE activity and severity are correlated to dysbiotic changes of the oral microbiota in periodontitis patients (25).

The prevalence, incidence and severity of periodontitis in SLE patients are controversial. Some studies show similar or better conditions in SLE compared to systemically healthy patients (26–28), while others suggested worst periodontal conditions in SLE (29–32). These conflicting results indicate that further research is necessary to better investigate the association between SLE and periodontitis.

The relevance and innovation of this study is that it investigates the presence of autoantibodies related to SLE in gingival tissue, which could contribute to the diagnosis of SLE. As a secondary goal, we aimed at investigating the prevalence, extension and severity of periodontitis in SLE patients compared to systemically healthy ones.

## Materials and Methods

### Subjects

SLE subjects were recruited at Lauro de Souza Lima Hospital and Bauru State Hospital from November 2017 to July 2019. It were included in the test group (SLE+) 25 female 20–65 years diagnosed with SLE according to ACR 1982/1997 revised classification criteria by a rheumatologist specialist. All patients were, at the moment of inclusion, in monitoring and/or treatment of SLE at one of the two Hospitals where the study was carried on. Control group (SLE–) was composed by 25 systemically healthy age-matched women, recruited at the Clinics of Periodontics at School of Dentistry at Bauru-USP during the same time period. It were excluded from the study patients with other autoimmune and/or rheumatological disorders (e.g., rheumatoid arthritis, Sjögren syndrome, pemphigoid, lichen planus), diabetics, pregnant women, presence of fixed orthodontic appliances, use of antibiotics in the 6-month period previous to inclusion, previous periodontal treatment (<12 mo.), edentulous, <8 teeth remaining, chronic renal failure requiring dialysis or diagnosis of malignant neoplasms <5 years.

### SLE Status

SLE diagnosis followed the guidelines defined by revised American College of Rheumatology criteria (6, 7). SLE activity was investigated by Systemic Lupus Erythemathosus Disease Activity Index (SLEDAI) (33). SLE inactivity was defined by SLEDAI ≤2 or by stable medications dose for at least 3 months and/or by daily prednisone dose <10 mg (8, 34). SLE activity was defined by SLEDAI >2 or daily prednisone doses >10 mg (8, 35). Disease severity was measured according to SLICC/ACR-DI (Systemic Lupus International Collaborating Clinics of American College of Rheumatology Damage Index) (35) to further characterize the studied population. SLE patients were subdivided into two groups based on the disease activity: active (SLE-A) or inactive (SLE-I). Antibodies anti-dsDNA were detected by immunofluorescence by the use of *Crithida luciliae* as substrate.

### Clinical Examinations

All participants have answered a health questionnaire to investigate medical and dental history. A visual examination of oral cavity was performed with the aid of disposable spatulas to investigate lesions at internal and external portions of lower and upper lips, cheek mucosa, retromolar area, tongue, oral floor, hard and soft palate, isthmus of the faces and upper and lower gingival mucosas.

Clinical periodontal examination was performed by a single trained examiner (JRP) by using a UNC-15 millimeter periodontal probe at six sites/tooth according to: pocket probing depth (PD), clinical attachment loss (CAL) and full-mouth bleeding scores (FMBS). Gingival recession (REC) was also determined at six sites/tooth as the distance from cementum-enamel junction (CEJ) to gingival margin. Full mouth plaque scores (FMPS) were assayed in four sites/tooth after visual inspection. Bone loss was determined in extra-oral digital panoramic X-ray images (1:1) as the distance from CEJ to alveolar bone crest (CEJ-AC) at mesial and distal sites of the tooth with the worst clinical periodontal condition (36).

All participants underwent non-surgical periodontal therapy before biopsy collection. Treatment consisted of supra and subgingival scaling and root planing, dental prophylaxis, oral hygiene instruction, elimination of plaque retention factors (open cavities, overhanging restorations, hopeless teeth), control of traumatogenic forces and splinting of mobile teeth.

### Histopathological and Direct Immunofluorescence Analysis

Incisional biopsies were obtained from gingival margin of 15 LES+ and 15 LES– patients requiring surgical periodontal treatment or for differential diagnosis of desquamative gingivitis lesions. All surgeries were performed by a single trained operator (JRP) under local anesthesia at Lauro de Souza Lima Hospital Dental Clinics (LES+) or at the Clinics of Periodontics, School of Dentistry at Bauru (LES–Samples were kept in cold saline solution for transfer to the laboratory, included in freezing medium, wrapped in aluminum foil and stored at −80°C until processing, which was performed at the Pathology Laboratory of Lauro de Souza Lima Hospital (Bauru, Brazil) by two pathologists (AJFN and MRSN).

Fragments were unfreeze and included in in glycolic resin embedding medium for cryotomy (Tissue-Tek, Sakura Finetek Europe BV, Netherland). Sections 5 μm-thick were obtained by using a cryostate (Leica CM1850, Leica Biosystems, Buffalo Grove, USA) at −22°C. Seven slides were obtained from each sample, each one containing two sections.

One section was stained by hematoxilin-eosin for descriptive histopathological analysis. The other section was prepared for direct immunofluorescence assay. Sections were delimited with a thin trace of varnish. After that, 100 μL of rabbit polyclonal FITC-conjugated antibodies (BioSB, Santa Barbara, CA, USA) against IgM, IgG, IgA, C1q, C3c diluted at 1:20 and F1b diluted at 1:80 were pipetted on the sections. Samples were incubated in humid and dark chamber for 30 min at 37°C, followed by washing in PBS and mounted in glass coverslips with 0.1% Evans Blue glycerin solution. Slides were wrapped in aluminum fail and kept at −20°C until the beginning of immunofluorescence reaction. After that, slides were kept in refrigerator at 4°C until analysis by fluorescence microscope (Zeiss Axioplan 2, Carl Zeis Microscopy, São Paulo, Brazil). The immunofluorescence reaction was classified semiquantitatively according to the intensity, varying from 1 to 3 (+, ++, +++).

### Statistical Analysis

Statistical analysis was performed at GraphPad Prism 8.0 for Mac software, at a 5% significance level for all analysis. Comparisons between groups were performed by Mann Whitney since a non-normal distribution was observed by Kolmogorov-Smirnov test. The association between systemic and oral conditions was investigated by Chi-square test. Histopathological findings were qualitatively described. The results from direct immunofluorescence were analyzed by Mann Whitney and Fischer exact test.

## Results

**Figure 1:**
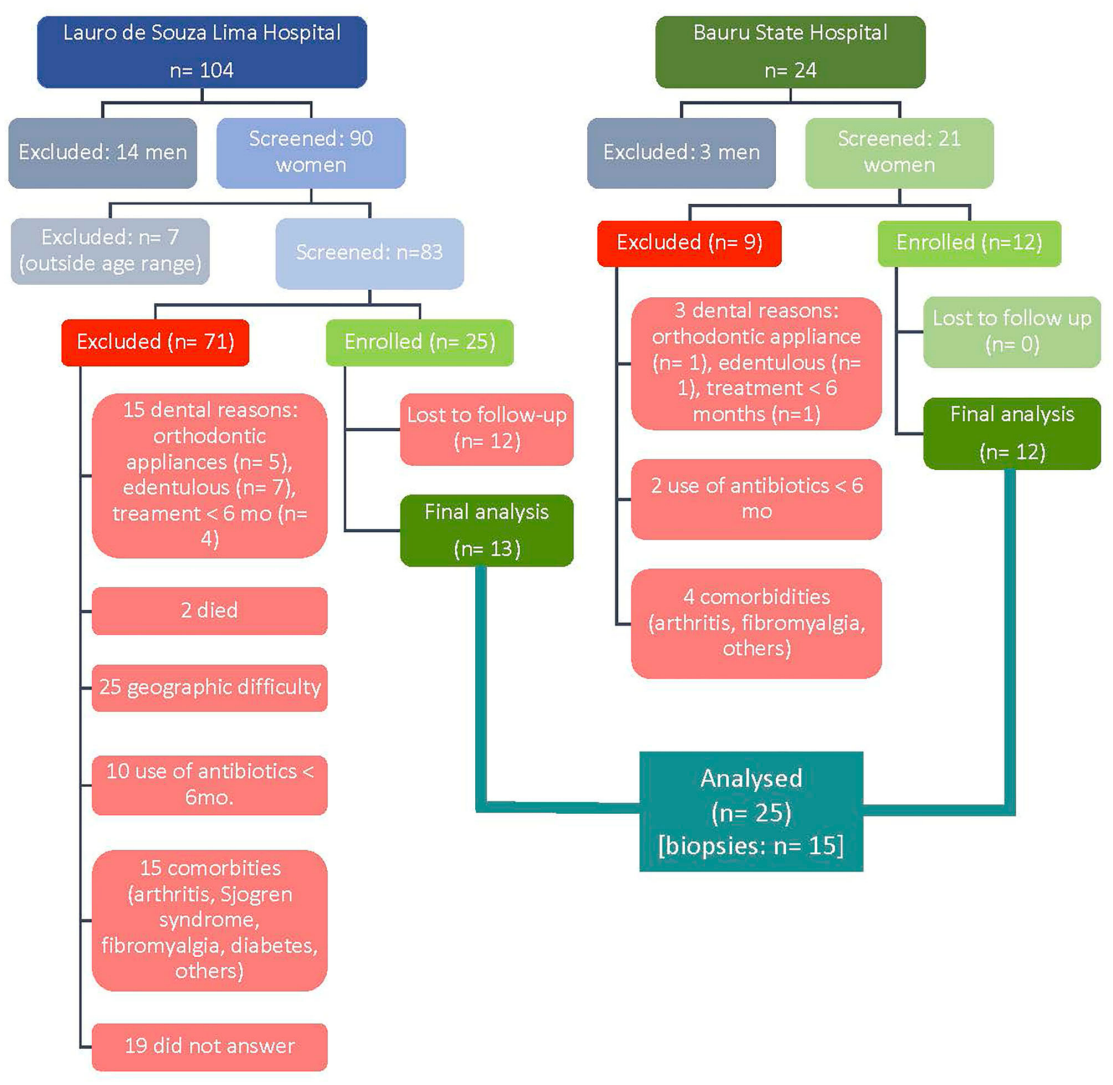
Epidemiological diagram of flux (LES+).

A total of 104 medical records of women diagnosed with SLE at the Medical and Statistical Archive Service of Lauro de Souza Lima Hospital were screened for inclusion in this study. From that, 13 women showing SLE only were included. The remaining 12 women included in test group were recruited at Bauru State Hospital, according to the same inclusion/exclusion criteria. Participants of the control group gender- and age-matched were recruited at School of Dentistry at Bauru and consecutively included in the study (Figure 1). The overall characteristics of SLE+ and SLE– groups are described in Table 1.

**Table 1:** Overall characteristicas of the sample.

	**SLE+ (*n* = 25)**	**SLE– (*n* = 25)**	***p*-value**
**Age**			
Age [mean (sd)]	41.34 (12.39)	43.73 (14.04)	0.53^**^
**Body mass index**			
BMI [mean (sd)]	27.46 (5.95)	26.60 (5.32)	0.58^**^
**Race**			
Caucasians [*n* (%)]	16 (64)	22 (88)	0.08^+^
Brown [*n* (%)]	6 (24)	3 (12)	
Black [*n* (%)]	3 (12)	0 (0)	
**Smoking**			
Smoker (*n* %)	3 (12)	0 (0)	0.20^+^
Former smoker (*n* %)	4 (16)	5 (20)	
Non-smoker (*n* %)	18 (72)	20 (80)	
**Medications**			
Oral contraceptives (*n* %)	4 (16)	6 (24)	0.47^+^
Antihypertensive^❖^ (*n* %)	2 (8)	4 (16)	0.38^+^

** *T-test; significant if p < 0.05*;

+ *Chi-square; significant if p < 0.05*;

❖ *Hydrochlorothiazide; sd- standard deviation; n-absolute number; %–percentage*.

### Characteristics of Active and Inactive SLE

Eleven patients (44%) had active (SLE-A) and 14 inactive (SLE-I) lupus. Characteristics of the groups are described in Table 2. No respiratory disorders were observed at SLE-A, which maximum daily dose of prednisone was higher than SLE-I to control flare. SLE-A showed greater circulating titers of anti-dsDNA and lower C4. The frequency of disease active descriptors in both groups is presented as Supplementary Table 1.

**Table 2:** Overall characteristics of LES-A and LES-I.

	**LES-A**	**LES-I**	**Valor do p**
Age (mean ± sd)	38.18 ± 12.26	43.82 ± 12.36	0.31^**^
Gender: female (*n* %)	11 (100%)	14 (100%)	–
Ethnicity [*n* (%)]			0.23^+^
White	5 (45.45)	11 (78.58)	
Mixed	4 (36.36)	2 (28.58)	
Black	2 (18.19)	1 (7.14)	
Central nervous system disease (*n* %)	2/8 (25)	0/5 (0)	0.15^+^
Kidney disease (*n* %)	5/8 (62.5)	3/7 (42.86)	0.44^+^
Respiratory disorders (*n* %)	0/8 (0)	3/7 (42.86)	**0.03**^+^
Cardiovascular diseases (*n* %)	4/8 (50)	3/7 (42.86)	0.78^+^
Gastoenteric disease (*n* %)	1/8 (12.50)	1/7 (14.29)	0.91^+^
**Comorbiditis (*****n*** **%)**			
Overweight or obesity (IMC ≥25)	4/11 (36.36)	10/14 (71.43)	0.07^+^
Hipertension	4/8 (50)	3/7 (42.86)	0.78^+^
Smoking (smokers and former smokers)	2/11 (18.18)	5/14 (35.71)	0.33^+^
**Medications in use**			
Prednisone dosage (mg/day) [mean ± sd]	18.64 ± 17.76	3.57 ± 3.63	0.049^**^
Hidroxicloroquine (*n* %)	11/11 (100)	11/14 (78.57)	0.10^+^
Antihypertensive (*n* %)	2/11 (18.18)	6/14 (42.86)	0.18^+^
Antidepressants, anxyolitics (*n* %)	1/11 (9.09)	4/14 (28.57)	0.22^+^
Imunossupressants (*n* %)	1/11 (9.09)	4/14 (28.57)	0.22^+^
Chemotherapy (*n* %)	2/11 (18.18)	1/14 (7.14)	0.39^+^
**Immuno-inflammatory response**			
High anti-dsDNA titers (*n* %)	5/8 (62.5)	0/5 (0)	**0.02**^+^
Low C4 (*n* %)	5/8 (62.5)	0/5 (0)	**0.02**^+^
Low C3 (*n* %)	3/8 (37.5)	1/5 (20)	0.50^+^
SLEDAI (mean ± sd)	11.0 ± 8.42	0.4 ± 0.89	0.005^**^

*Medical history and laboratory tests are not available for the entire sample; available data (number of positive cases / total cases (%)*;

** *t-test*;

+ *Chi-Square Test; significant if p <0.05; sd, standard deviation. Bold values - significant differences between groups*.

### Periodontal Parameters

Mean PD, CAL and FMBS were significantly greater in SLE– women than in SLE+, with no significant differences observed in FMPS, CEJ-AC and tooth loss. Additionally, a greater percentage of sites with PD ≥5 mm was observed in SLE– (Table 3). Periodontitis was diagnosed in 22 (88%) of SLE+ and in 20 (80%) of SLE– participants, with no differences between groups. There was a prevalence of Stage III periodontitis, affecting 17 (68%) and 18 (72%) of SLE+ and SLE–, respectively. Most patients were assigned as Grade B, with no differences between SLE+ (52%) and SLE– (44%). Additionally, extension of periodontitis lesion was <30% (localized) in 86.36% of SLE-A and in 60% of SLE– participants. More detail in Supplementary Table 2.

**Table 3:** Periodontal parameters observed in SLE+ and SLE– women.

	**SLE+**	**SLE–**	***p*-value**
**REC**			
mean (sd)	0.15 (0.19)	0.22 (0.39)	0.41^*^
median (95% CI)	0.11 (0.07; 0.23)	0.05 (0.06; 0.39)	
**PD**			
mean (sd)	2.18 (0.55)	2.87 (1.06)	0.01^*^
median (95% CI)	2.06 (1.95; 2.41)	2.67 (2.44; 3.31)	
**CAL**			
mean (sd)	2.34 (0.53)	3.07 (1.14)	0.005^*^
median (95% CI)	2.19 (2.12; 2.56)	2.79 (2.60; 3.54)	
**FMBS**			
mean (sd)	26.86 (16.14)	49.53 (33.78)	0.01^*^
median (95% CI)	23.89 (20.19; 33.52)	50 (35.59; 63.48)	
**FMPS**			
mean (sd)	49.53 (22.16)	0.53 (0.35)	0.46^*^
median (95% CI)	50 (40.39; 58.68)	61.46 (39.35; 68.53)	
**CEJ-AC**			
mean (sd)	1.37 (0.68)	1.23 (0.52)	0.26^*^
median (95% CI)	1.24 (1.08; 1.64)	1.14 (1.02; 1.45)	
**Missing teeth**			
mean (sd)	7.68 (5.44)	8.28 (5.18)	0.69^*^
median (95% CI)	6 (5.43; 9.92)	6 (6.14; 10.42)	
**% sites PD** **≥5 mm**			
median (95%CI)	1.23 (1.51; 6.35)	6.94 (6.97; 19.95)	0.002^*^
**% sites CAL** **≥4 mm**			
median (95%CI)	7.14 (7.99; 16.74)	22.22 (17.36; 35.69)	0.06^*^

* *Mann Whitney; significante se p <0.05. Bold values - significant differences between groups*.

The association between periodontal parameters and systemic conditions was investigate by Chi-squared bivariate analysis. CAL was significantly lower in patients using low daily doses of prednisone (≤10 mg/day), suggesting a beneficial effect of the chronic use of immunosuppressants in periodontal attachment loss (Table 4). Details on the characteristics of drug therapy for SLE+ are presented in Supplementary Table 3.

**Table 4:** Association between daily doses of prednisone and periodontal parameters.

	**>10 mg/day**	**≤ 10 mg/day**	***p* value**	**OR (95% CI)**
PD > 2 mm	3 (12%)	9 (36%)	0.91	1.11 (0.21; 5.72)
PD ≤ 2 mm	3 (12%)	10 (40%)		
CAL > 3 mm	2 (8%)	1 (4%)	**0.03**	12.67 (1.03; 189.6)
CAL ≤ 3 mm	3 (12%)	19 (76%)		
FMBS ≥ 30%	1 (4%)	7 (28%)	0.74	0.66 (0.04; 5.30)
FMBS <30%	3 (12%)	14 (56%)		
CEJ-ABC > 2	1 (4%)	1 (4%)	0.36	3.6 (0.15 – 71.43)
CEJ- ABC ≤ 2	5 (20%)	18 (72%)		

*Chi-square test; significant if p <0.05. Bold values - significant differences between groups*.

### Histopathological Analysis

**Figure 2:**
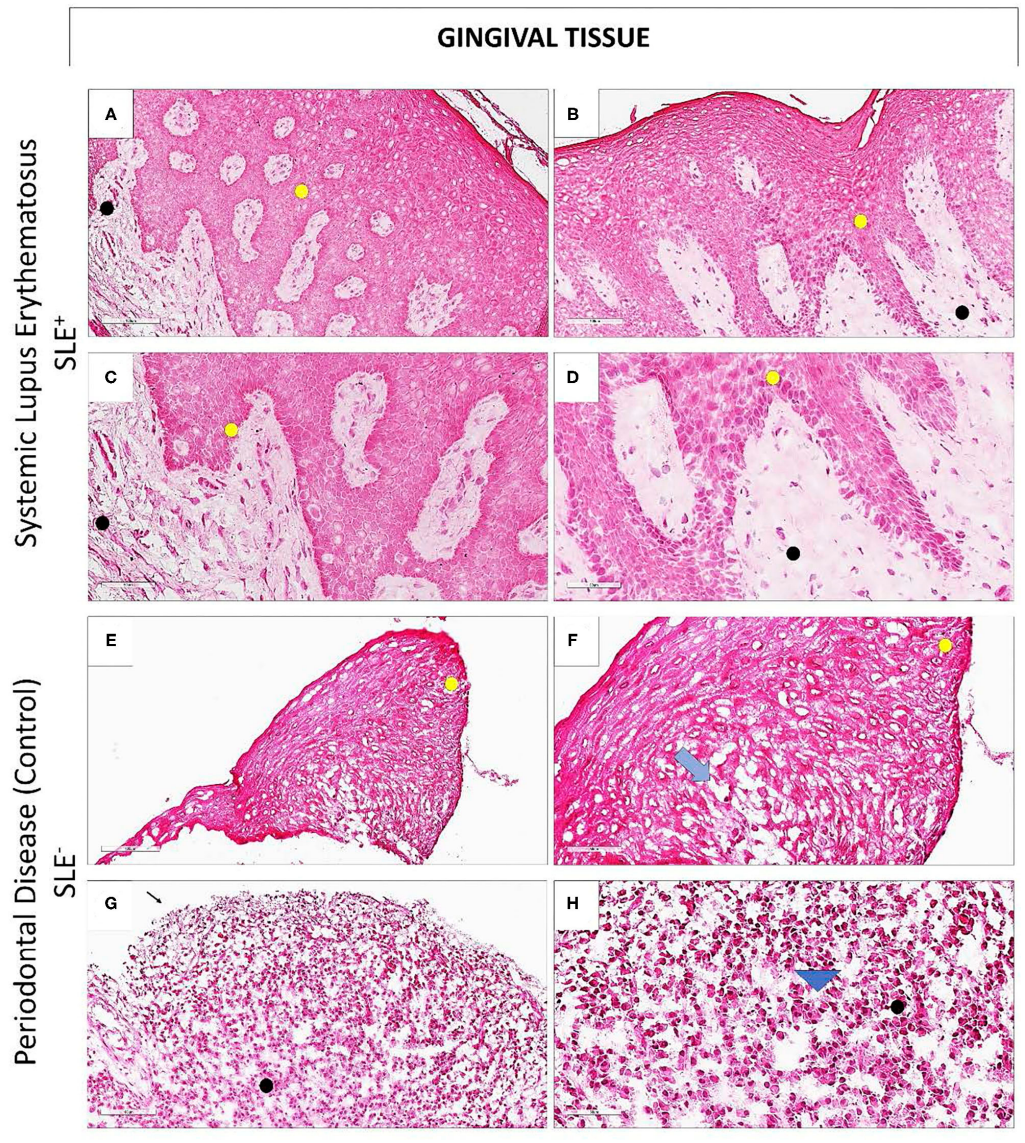
Histopathological findings of gingival biopsies obtained from SLE+ and SLE– women. Microscopic section of the region between the free and attached gingiva of a patient with periodontitis and SLE (**A–D**; SLE+) and a systemically healthy, periodontitis patient (**E–H**; SLE–) **(A,B)**. Note that the gingival mucosa remains healthy, even in the presence of epithelial hiperplasia **(C,D)**; no dysplastic or structural changes in the layers of the epithelium (yellow circle) or in the lamina propria (black circle) were noticed **(E,F)**. In SLE–, periodontitis patient, it is noticed the onset of exocytosis (**F**; blue arrow), and areas of epithelial ulceration **(G)**. A dense inflammatory infiltrate composed mainly by lymphocytes and plasma cells occupying >50% of gingival connective tissue area is noticed (H; arrowhead) (**A,B** and **E,F**: 100μm magnification; **C,D** and **G,H**: 50μm magnification).

Gingival biopsies were obtained from 13 SLE+ diagnosed with periodontitis, 2 SLE+ with no periodontitis, and 15 SLE– with periodontitis. Histopatologic findings from SLE+ showed minimal mononuclear inflammatory infiltrate in the chorion, predominantly perivascular, with no significant morphological changes in the epithelium and intact basement membrane (Figures 2A–D). Histologic characteristics of LES- showed a dense inflammatory infiltrate predominantly composed by lymphocytes and plasma cells, occupying a vast area of gingival connective tissue (Figures 2E–H).

### Direct Immunofluorescence

**Figure 3:**
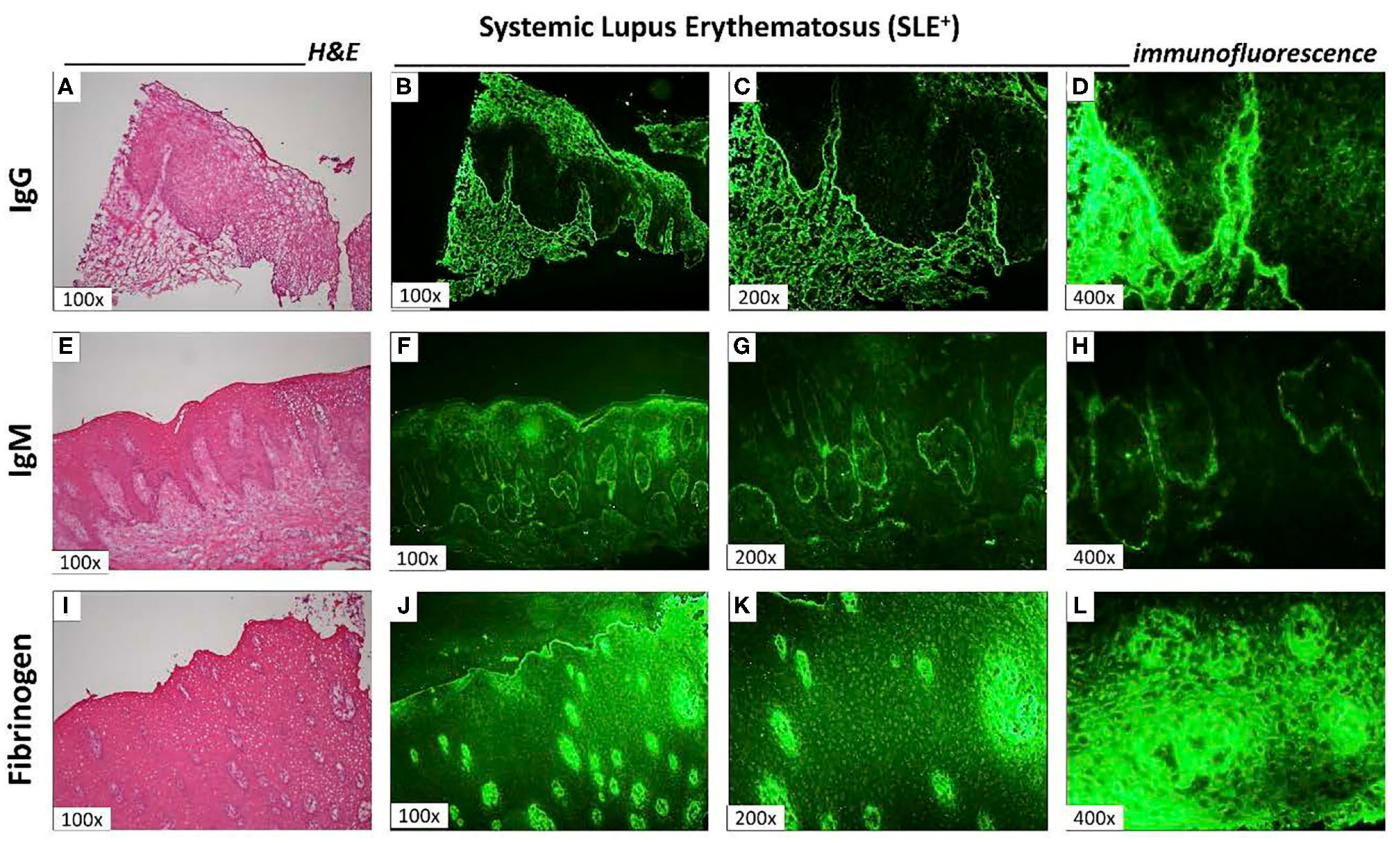
Direct immunofluorescence at LES+ **(A,E,I)**. Hematoxylin and eosin sections obtained from three different SLE+ patients to confirm histopathological diagnosis (100x magnification); Direct immunofluorescence to analyze the presence of IgG (**B** 100x; **C** 200x; **D** 400x magnification of gingival biopsy shown in **A**); IgM (**F** 100x; **G** 200 x; **H** 400 x magnification of gingival biopsy shown in **E**) and fibrinogen (**J** 100x; **K** 200x; **L** 400x magnification of section shown in **I**). The immunoglobulin immunostaining was granular in appearance, focusing mainly on areas of loose connective tissue near the basement membrane. However, the detection of fibrinogen has also been found in epithelial cells **(L)**.

Direct immunofluorescence was performed on all biopsies (*n* = 15/group) with anti-IgG, anti-IgM, anti-IgA, anti-C1q, anti-C3c (LN markers) and anti-fibrinogen (F1b) FITC-conjugated antibodies. SLE + patients demonstrated immunopositivity for IgG (Figures 3A–D) and IgM (Figures 3E–H) antibodies, mainly in the loose connective tissue regions permeating the epithelial ridges. Fibrinogen was homogeneously expressed in the connective tissue of SLE+ patients (Figures 3I–L). Additionally, SLE+ showed immunopositivity for IgA and C3c. The anti-C1q antibody was not reactive to immunofluorescence in any patient of both groups. In SLE, immune complexes IgG, IgM, IgA, and C3c were directed against basement membrane.

Quantitative analysis of direct immunofluorescence is shown in Table 5. Only 1 SLE– patient showed the presence of LN markers in gingival biopsies, while 9 SLE+ showed LN markers (*p* = 0.005; OR = 21; 2.46; 242.9; Fischer Exact Test). Comparisons between groups showed significant more labeling of IgM in SLE+ than SLE–. Both groups showed antibodies anti-F1q in the connective tissue. Detailed information is presented in Supplementary Table 4.

**Table 5:** Quantitative analysis of direct immunofluorescence.

	**SLE+ [median; (mean ± sd)]**	**SLE– [median; (mean ± sd)]**	***p*-value**
IgG	0 (0.13 ± 0.51)	0 (0)	>0.99
IgM	0 (0.60 ± 0.82)	0 (0.06 ± 0.25)	**0.03**
IgA	0 (0.06 ± 0.25)	0 (0)	>0.99
C1q	0 (0)	0 (0)	>0.99
C3	0 (0.20 ± 0.41)	0 (0.06 ± 0.25)	0.59
F1b	2 (1.46 ± 0.63)	1 (1.20 ± 0.41)	0.12
Total	2 (2.46 ± 1.55)	1 (1.33 ± 0.81)	**0.01**

* *Mann Whitney; significant if p <0.05. Bold values - significant differences between groups*.

## Discussion

The diagnosis of SLE is complex and relies on the presence of four signs and symptoms, necessarily including one clinical and one laboratorial finding or the biopsy-proven lupus nephritis (LN) (1). We have used antibodies against IgM, IgG, IgA, C1q and C3c, frequently used for the diagnosis of LN by direct immunofluorescence, to investigate the deposition of immune complexes in the gingival connective tissue in SLE patients compared to systemically healthy ones, both with periodontitis. Our results showed the presence of immune complexes in gingiva of nine out of 15 SLE+ patients and in one out of 15 SLE– patients, with significant differences between groups (*p* = 0.005; Fischer Exact test). From the panel of antibodies investigated, IgM was detected in seven SLE+, with weak (+) to strong (+++) scores, IgG and IgA were detected in one patient each and C3c was detected in 3 patients, compared to only one patient presenting immunostaining against IgM and C3c in only one SLE– patient (Supplementary Table 4).

This finding might aid in the diagnosis of lupus, which is quite difficult to achieve due to the variability and complexity of clinical signs and symptoms, which may also manifest in diseases or conditions others than lupus (1).

The formation of immune complexes is a typical finding in autoimmune diseases. It may arise from circulating antibodies or be formed by the reaction of immunoglobulins with cell or tissue antigens or to bacteria temporarily adsorbed to cells, in a type III hypersensitivity reaction (37). In autoimmune diseases, such as SLE, these reactions typically occur in a granular pattern at the basement membrane (Figure 3), as observed in our study. Besides immunoglobulins and complement components, we have also investigated F1b, which was observed in 100% of SLE– and in 93.3% of SLE+ (Supplementary Table 4). This marker is associated with revascularization, wound healing and tissue repair and may also be expressed in different disorders, such as chronic kidney disease or amyloidosis.

Deposition of immune complexes in lupus are usually higher at the diagnosis. The presence of lupus-associated autoantibodies may exist in apparently healthy individuals (38), some of which in the pre-clinical phase (1). Antibodies titers and types vary depending on the stage of development of the disease. In the pre-clinical phase, 25% of the population is ANA positive in titers >1:40, 5% in titers ≥1:160 and 2% in titers considered as pathological (39). On the other hand, more than 99% of SLE patients are ANA-positive at some point during the course of the disease, besides the presence of other circulating autoantibodies, such as anti-Ro, anti-La, anti-phospholipids, anti-RNP and anti-dsDNA years before the clinical manifestation of SLE (1). The number of specific antibodies gradually increases in SLE up to the moment of diagnosis, and its accumulation decreases thereafter (40). Three phases are observed in disease development: (1) normal phase in asymptomatic individuals without SLE antibodies; (2) benign immunity, under the influence of genetic and environmental factors, characterized by the presence of autoantibodies (ANA, anti-Ro, anti-La or anti-aPL) in peripheral blood vessels in the absence of clinical manifestations; (3) pathogenic autoimmunity, characterized by the presence of anti-dsDNA, anti-Sm and anti-RNP and the development of clinical signs and symptoms (1).

Our study included women with established diagnosis and in treatment of SLE. Therefore, in these patients, deposition of autoantibodies in organs and tissues had already taken place. Even so, the presence of higher levels of immunoglobulins and complement in the basement membrane of SLE+ periodontitis patients, especially IgM, provides evidence of the disease, and might be helpful for the diagnosis of SLE or its flare. Higher titers of circulating antibodies, low C3, C4 and CH50 and increased deposition of immune complexes in tissues are seen during lupus activity, especially on those who develop LN, tending to normalize with clinical improvement (9, 41, 42).

Patients with active kidney disease tended to have lower levels of CH50 and C3 and higher levels of immune complexes detected by C1qBA than those with extra-renal manifestations only. Patients with renal and extrarenal manifestations have lower levels of CH50, C4 and C3, but the deposition of immune complexes in such cases is lower than those observed in LN. These findings highlight the concept that SLE and LN are two autoimmune conditions characterized by isotype specificity of auto-antibodies (42). In our sample, 5 patients had renal or extra-renal diseases and, from that, 2 presented IgM immunostaining and 1 C3. No patient showed positive staining for C1q in neither groups (Supplementary Table 4). Low levels of complement without high levels of C1q suggest unlikely kidney disease (9). Additionally, it could be observed that SLE-A patients showed significantly higher titers of anti-dsDNA and lower C4 levels than SLE-I (Table 2), corroborating these findings.

Different studies showed increased serum IgG titers against periodontal pathogens in chronic periodontitis (16, 18–20) and even higher titers in gingival crevicular fluid (43). Immune complexes and IgG deposits with active complement factors were noticed in periodontitis patients (15, 17), associated with increased number of osteoclasts in alveolar bone crest, suggesting their involvement in the acute phase of periodontal destruction (21).

Histopathological findings of gingival biopsies obtained from SLE+ patients showed minimal amount of inflammatory infiltrate at gingival connective tissue in periodontitis patients (Figure 2), contrasting with the findings of SLE– periodontitis patients, who showed a dense infiltrate predominantly composed of lymphocytes and plasma cells (44). This can be explained by the use of immunosuppressants or immunomodulators for lupus control. Only one SLE-I did not use any of these drugs (Supplementary Table 3). A positive association between low daily doses of prednisone (<10 mg) and CAL ≤ 3 mm was observed (Table 4). This finding emphasizes the role of immune inflammatory host responses in the pathogenesis of periodontitis.

The suppression or modulation of immune inflammatory host response correlates with clinical periodontal parameters. Worse periodontal conditions, especially PD, CAL, FMBS and percentage of sites with PD ≥5 mm, were observed in SLE– than in SLE+ women (Table 3), in spite of similar overall characteristics of SLE+ and SLE– (Table 1). These findings differ from other studies which showed worse periodontal conditions in SLE patients compared to healthy ones (31, 45, 46). Gofur et al. (47) showed that worst periodontal conditions were associated with higher SLEDAI scores. However, the maximum SLEDAI in our sample was 28 (data not shown), which may account for differences observed between our and other studies. Other reports, however, did not find differences in periodontal parameters of SLE patients (27, 45, 48).

No differences in the prevalence of healthy/periodontitis patients were observed in SLE+ and SLE- (Supplementary Table 2). In SLE+, 2 patients (8%) and 1 (4%) were classified as periodontally healthy or with gingivitis, respectively, according to 2018 AAP/EFP classification of periodontal and peri-implant diseases and conditions (36). In SLE–, five patients (20%) were classified as healthy, with no significant differences between groups. A recent systematic review and meta-analysis (49) including eight case-control studies with 487 cases of SLE and 1383 participants in total, found that the risk of periodontitis in cases of SLE was significantly higher than in systemically healthy controls, with RR of 1.76 (95% CI 1.29–2.41; *p* = 0.0004). However, no significant differences were observed between groups in relation to periodontal measures, such as probing depth and loss of clinical insertion, as also observed in our study.

Pessoa et al. (25) evaluated the reciprocal impact of the subgingival microbiota on systemic inflammation in patients with SLE. Ninety-one women were recruited, 31 of whom were systemically healthy, 29 with inactive SLE and 31 with active SLE. There was a high expression of pro-inflammatory cytokines in patients with SLE compared to healthy controls. In SLE-I, low-intensity inflammation was observed, while a potent anti-inflammatory cytokine, IL-10, attenuated clinical phenotypes. Of 24 significant oral microbial abundances found in patients with SLE, 14 unique subgingival bacterial profiles were elevated in SLE, with a particular increase in the levels of *T. denticola* and *T. forsythia* in patients with SLE-A compared to control. The cytokine-bacteria correlations The correlation between cytokines and bacteria showed that periodontal pathogens dominating the environment increased systemic levels of cytokines. Deeper bags and greater loss of insertion were observed in SLE patients, especially SLE-I, possibly due to chronic, long-lasting, low-intensity inflammation. Thus, taking into account the results of another study (45), it can be hypothesized that, although there is dysbiosis in patients with SLE, the clinical manifestation of periodontitis in these patients is masked by the use of corticosteroids and immunosuppressants.

As far as we know, no study has investigated lupus-related deposition of antibodies in gingival tissues. Additionally, to date, no serum or urine biomarker is sufficiently accurate in the diagnosis of incipient or recurrent LN so that renal biopsies could be replaced (50, 51). Our findings, although interesting, should be further investigated, since all patients were diagnosed and in long-term treatment for active or inactive lupus, which might contribute not only to decreased deposition of immune complexes at gingival tissues as well as to better clinical parameters in test group. Considering so, further studies are necessary to confirm or discard our hypothesis that lupus-associated immune complexes deposit in gingival tissues and may aid in the diagnosis of the disease or its flare.

The findings of this study suggest that lupus-associated immune complexes can be detected by direct immunofluorescence in biopsies of gingiva, which could aid in diagnosis of the disease. Besides that, the use of immunosuppressants and immunomodulators limits density and extension of the inflammatory infiltrate at gingival tissues, contributing to a better clinical periodontal condition.

## Data Availability Statement

The original contributions presented in the study are included in the article/Supplementary Material, further inquiries can be directed to the corresponding author/s.

## Ethics Statement

The studies involving human participants were reviewed and approved by School of Dentistry at Bauru – USP Ethics Committee. The patients/participants provided their written informed consent to participate in this study.

## Author Contributions

AS, LP, and SG conceived and designed the experiments. JP performed the experiments and tabulated data. MN, AN, and DD performed histopathologic and direct immunofluorescence assays. MZ, CD, and MR contributed in control group treatment. AS, JP, and LP wrote the paper. All authors contributed to the article and approved the submitted version.

## Conflict of Interest

The authors declare that the research was conducted in the absence of any commercial or financial relationships that could be construed as a potential conflict of interest.
