# Supplementary material for: Deposition of Immune Complexes in Gingival Tissues in the Presence of Periodontitis and Systemic Lupus Erythematosus
Source: Front Immunol. 2021 Mar 25;12:591236. doi: 10.3389/fimmu.2021.591236 (PMC8027066; doi:10.3389/fimmu.2021.591236)
Supplement: Supplementary file 1 [file Table_1.docx]

**Supplementary Table 1** – Frequency of SLEDAI descriptors at SLE-A and SLE-I

|  | LES-A | LES-I | p |
| --- | --- | --- | --- |
| *Vasculitis (n, %)* | 1/8 (12,5) | 0/5 (0) | 0,41^+^ |
| *Myositis (n, %)* | 0/5 (0) | 1/8 (12,5) | 0,18^+^ |
| *Urine cylinders (n, %)* | 1/8 (12,5) | 0/5 (0) | 0,41^+^ |
| *Hematuria (n, %)* | 4/8 (50) | 0/5 (0) | 0,05^+^ |
| *Proteinuria (n, %)* | 6/8 (75) | 0/5 (0) | **0,008^+^** |
| *Pyury (n, %)* | 1/8 (12,5) | 0/5 (0) | 0,41^+^ |
| *Malar rash (n, %)* | 2/8 (25) | 0/5 (0) | 0,22^+^ |
| *Oral or nasal ulcers (n, %)* | 1/8 (12,5) | 0/5 (0) | 0,41^+^ |
| *Pleuritis (n, %)* | 1/8 (12,5) | 0/5 (0) | 0,41^+^ |
| *Platelets < 100.000/mm^3^ (n, %)* | 0/8 (0) | 0/5 (0) | -- |
| *Leucopenia < 3000/mm^3^ (n, %)* | 0/8 (0) | 0/5 (0) | -- |

Obs.: There was no report of seizure, psicoses, organic cerebral syndrome, visual disturbes, cranial nerve disturbes, lupus headache, stroke, arthritis, alopecia, pericarditis or fever > 38^o^C.

^+^Chi-squared; significant if p< 0.05
