# Supplementary material for: Deposition of Immune Complexes in Gingival Tissues in the Presence of Periodontitis and Systemic Lupus Erythematosus
Source: Front Immunol. 2021 Mar 25;12:591236. doi: 10.3389/fimmu.2021.591236 (PMC8027066; doi:10.3389/fimmu.2021.591236)
Supplement: Supplementary file 2 [file Table_2.docx]

**Supplementary Table 2** – Classification of periodontal diseases in SLE+ and SLE-

|  |  | SLE + | SLE- | p-value |
| --- | --- | --- | --- | --- |
| Classification | | | | |
| Health | Yes  No | 2 (8%)  23 (92%) | 5 (20%)  20 (80%) | 0.41 |
| Gingivits | Yes  No | 1 (4%)  24 (96%) | 0 (0%)   1. (100%) | > 0.99 |
| Periodontitis | Yes  No | 22 (88%)  3 (12%) | 20 (80%)  5 (20%) | 0.70 |
| Staging | | | | |
| Stage II | Yes  No | 4 (16%)  21 (84%) | 0 (0%)  25 (100%) | 0.10 |
| Stage III | Yes  No | 17 (68%)  8 (32%) | 18 (72%)   1. (28%) | > 0.99 |
| Stage IV | Yes  No | 1 (4%)  24 (96%) | 2 (8%)  23 (92%) | > 0.99 |
| Grading | | | | |
| Grade A | Yes  No | 5 (20%)  20 (80%) | 6 (24%)  19 (76%) | > 0.99 |
| Grade B | Yes  No | 13 (52%)  12 (48%) | 11 (44%)  14 (56%) | 0.77 |
| Grade C | Yes  No | 4 (16%)  21 (84%) | 3 (12%)  22 (88%) | > 0.99 |
| Distribution | | | | |
| Extent | < 30%  ≥ 30% | 19 (86,36%)  3 (13,64%) | 12 (60%)  8 (40%) | 0.08 |

<30% of teeth: localized periodontitis; ≥ 30% of teeth: generalizaed periodontitis. Chi-squared test; p-value: significant if p<0.05. There was no Stage I periodontitis.
