# Supplementary material for: Deposition of Immune Complexes in Gingival Tissues in the Presence of Periodontitis and Systemic Lupus Erythematosus
Source: Front Immunol. 2021 Mar 25;12:591236. doi: 10.3389/fimmu.2021.591236 (PMC8027066; doi:10.3389/fimmu.2021.591236)
Supplement: Supplementary file 3 [file Table_3.docx]

**Supplementary Table 3 –** Drug regimens of SLE-A and SLE-I patients

| Code | Classification | Drug regimen | Immunological markers | SLEDAI/SLIIC criteria | Gingival biopsy (yes/no) |
| --- | --- | --- | --- | --- | --- |
| SLE001 | SLE-A | HCQ | ↑ anti-dsDNA  ↓ C4 | LN | Yes |
| SLE002 | SLE-I | PRD 10mg/day + HCQ + AZA | N/A | N/A | Yes |
| SLE003 | SLE-A | PRD 5mg/day + HCQ | Normal | Proteinuria | Yes |
| SLE004 | SLE-I | HCQ | N/A | N/A | Yes |
| SLE005 | SLE-I | ADP | Normal | LN | Yes |
| SLE006 | SLE-A | PRD 60mg/day + HCQ + RXM | ↓ C4 | Proteinuria  Pericarditis  LN | No |
| SLE007 | SLE-I | PRD 5mg/day + HCQ + MTX | N/A | N/A | Yes |
| SLE008 | SLE-I | HCQ | N/A | N/A | No |
| SLE009 | SLE-I | HCQ | N/A | None | Yes |
| SLE010 | SLE-A | PRD 20mg/day + HCQ | N/A | N/A | Yes |
| SLE011 | SLE-I | PRD 5mg/day + HCQ + AZA | N/A | N/A | Yes |
| SLE012 | SLE-I | PRD 10mg/day + AZA | ↓ C3 | None | Yes |
| SLE013 | SLE-I | HCQ | N/A | N/A | No |
| SLE014 | SLE-I | PRD 5mg/day | N/A | Hypertension  Kidney transplantation | Yes |
| SLE015 | SLE-I | PRD 5mg/day + HCQ + AZA | N/A | N/A | Yes |
| SLE016 | SLE-A | PRD 40mg/day + HCQ | ↑ anti-dsDNA  ↓ C3  ↓ C4 | Vasculitis Hematuria  Proteinuria  Pyury  Malar rash  Oral ulcers  Hypertension  LN  CNS disorders | No |
| SLE017 | SLE-I | HCQ | Normal | Respiratory | No |
| SLE018 | SLE-A | PRD 20mg/day + HCQ | ↑ anti-dsDNA  ↓ C3  ↓ C4 | Proteinuria  Hematuria  Malar rash  Pleuritis | Yes |
| SLE019 | SLE-A | PRD 20mg/day + HCQ | N/A | N/A | No |
| SLE020 | SLE-I | PRD 5mg/day + HCQ | Normal | Gastroenteric  Respiratory  Hypertension | Yes |
| SLE021 | SLE-A | PRD 5mg/day + HCQ + MTX | ↑ anti-dsDNA | Urinary cilinders  Hematuria  Proteinuria  Hypertension  LN | No |
| SLE022 | SLE-A | PRD 10mg/day + HCQ | ↑ anti-dsDNA  ↓ C3 | Hematuria  Gastroenteric | No |
| SLE023 | SLE-A | PRD 5mg/day + HCQ | Normal | CNS disorders | No |
| SLE024 | SLE-I | PRD 5mg/day + HCQ | Normal | Cardiovascular  LN | No |
| SLE025 | SLE-A | PRD 20mg/day + HCQ | N/A | N/A | Yes |

HCQ- hydroxychloroquine 400 mg/day; PRD- prednisone; AZA- azathioprine; ADP- antidepressants; RXM- rituximab endo-venous; MTX- methotrexate; LN- lupus nephritis; N/A- not available
