# Supplementary material for: Deposition of Immune Complexes in Gingival Tissues in the Presence of Periodontitis and Systemic Lupus Erythematosus
Source: Front Immunol. 2021 Mar 25;12:591236. doi: 10.3389/fimmu.2021.591236 (PMC8027066; doi:10.3389/fimmu.2021.591236)
Supplement: Supplementary file 4 [file Table_4.docx]

**Supplementary Table 4** – Characteristics of SLE+ and SLE- direct immunofluorescence assay

| ***SLE+*** | | | | | | | | | | | ***SLE-*** | | | | | | | |
| --- | --- | --- | --- | --- | --- | --- | --- | --- | --- | --- | --- | --- | --- | --- | --- | --- | --- | --- |
| **Age** | **Subgroup** | **Drug^*^** | **Perio^**^** | **USD^***^** | **DI Antibody** | | | | | | **Age** | **Perio^**^** | **DI Antibody** | | | | | |
|  |  |  |  |  | **IgG** | **IgM** | **IgA** | **C1q** | **C3** | **F1b** |  |  | **IgG** | **IgM** | **IgA** | **C1q** | **C3c** | **F1b** |
| 46,6 | SLE-A | 1 | IIIC | 1 | - | - | - | - | - | + | 48 | IIIB | - | - | - | - | - | + |
| 36,8 | SLE-I | 1 | IIIB | N/A | - | + | - | - | - | ++ | 58,4 | IIIA | - | - | - | - | - | + |
| 35,4 | SLE-A | 1 | IIIB | 1 | - | + | - | - | - | ++ | 55,7 | IIIA | - | - | - | - | - | + |
| 64,3 | SLE-I | 1 | IVA | N/A | - | - | - | - | - | + | 27,4 | IIIA | - | - | - | - | - | + |
| 29,4 | SLE-I | 0 | IIA | 1 | - | - | - | - | + | ++ | 48,9 | IVB | - | - | - | - | - | ++ |
| 44,7 | SLE-I | 1 | IIIC | N/A | ++ | + | - | - | + | ++ | 54 | IIIC | - | - | - | - | - | + |
| 59,4 | SLE-I | 1 | IIB | 0 | - | + | - | - | + | ++ | 61,4 | IIIB | - | - | - | - | - | + |
| 37,9 | SLE-A | 1 | IIIA | N/A | - | +++ | - | - | - | ++ | 54,7 | IVA | - | - | - | - | - | + |
| 34,4 | SLE-I | 1 | IIIC | N/A | - | - | - | - | - | + | 59,7 | IIIB | - | - | - | - | - | + |
| 36,3 | SLE-I | 1 | IIB | 0 | - | + | + | - | - | - | 57,2 | IIIC | - | - | - | - | - | + |
| 50,6 | SLE-I | 1 | 0 | 1 | - | + | - | - | - | + | 44,8 | IIIC | - | + | - | - | + | ++ |
| 58,8 | SLE-I | 1 | 0 | N/A | - | - | - | - | - | + | 60,8 | IIIB | - | - | - | - | - | ++ |
| 42,8 | SLE-A | 1 | IIIB | 1 | - | - | - | - | - | + | 58,7 | IIIA | - | - | - | - | - | + |
| 56,2 | SLE-I | 1 | IIIB | 0 | - | - | - | - | - | ++ | 40,5 | IIIB | - | - | - | - | - | + |
| 31 | SLE-A | 1 | IIIB | N/A | - | +++ | - | - | - | ++ | 61,7 | IIIB | - | - | - | - | - | + |

^*^use of immunosuppressants drugs: 1- yes; 0- no

^**^ Classification of periodontitis according to Tonetti et al., 2018 (Stage and Grade)

^***^ Urinary system disorders: 1- yes; 0- no; N/A- not available
